# Special Issue “Molecular Biology of Host and Pathogen Interactions: 2nd Edition”

**DOI:** 10.3390/ijms262110715

**Published:** 2025-11-04

**Authors:** Ana Paula Arez

**Affiliations:** Global Health and Tropical Medicine (GHTM), Associate Laboratory in Translation and Innovation Towards Global Health (LA-REAL), Instituto de Higiene e Medicina Tropical (IHMT), Universidade NOVA de Lisboa (UNL), Rua da Junqueira 100, 1349-008 Lisboa, Portugal; aparez@ihmt.unl.pt

## 1. Introduction

The complex interactions between hosts and pathogens represent one of the most dynamic and rapidly evolving areas of biological research, and recent years have witnessed remarkable advances in our understanding of the molecular mechanisms that govern host–pathogen interplay. Studies have clarified not only how pathogens invade, persist in, and sometimes hijack host systems, but also how hosts mount intricate defenses at genetic, metabolic, and cellular levels.

Recent and key developments in the field have been driven by technological advances in areas such as genomics, proteomics, transcriptomics, and advanced imaging techniques. The integration of genomic, transcriptomic, proteomic, and metabolomic approaches has provided unprecedented insights into the temporal and spatial dynamics of infection processes, revealing how pathogens orchestrate complex regulatory networks to establish infection and persist within hosts [1,2,3,4]. The advent of single-cell technologies has revolutionized our understanding of cellular heterogeneity during infection, revealing previously hidden subpopulations of cells with distinct responses to pathogens [5,6,7,8]. Advanced bioinformatics approaches such as network medicine and machine learning, mathematical modeling and systems-level analyses have enhanced our ability to predict interactions and infection outcomes and identify potential therapeutic targets [9,10,11].

## 2. Contributions of This Special Issue

The articles in this Special Issue, “Molecular Biology of Host and Pathogen Interactions: 2nd Edition” (see Table 1), address several knowledge gaps through diverse experimental approaches and pathogen systems. The nine contributions published in this collection span viral, bacterial, parasitic, and fungal pathogens, and offer new insights into the role of virulence factors and immune evasion tactics, the deployment of omics techniques and computational systems biology to construct models of these complex biological confrontations, the exploration of both traditional protein–protein interactions and emerging research areas such as non-coding RNAs, metabolic network interdependencies, and the systems-level modeling of immunological responses and development of novel research tools, experimental methods, and databases that facilitate the comprehensive analysis of pathogen–host interactions.

### 2.1. Hormonal Modulation of Host Responses

The role of sex hormones in modulating host–pathogen interactions remains underexplored [12,13]. Nolasco-Pérez et al. provide crucial insights into the sexually dimorphic nature of malaria pathogenesis, demonstrating how testosterone modulates oxidative stress responses during *Plasmodium berghei* infection in mice. This work addresses the significant gap in our understanding of sex-specific factors in infectious disease outcomes and highlights the importance of considering hormonal influences in therapeutic strategies.

### 2.2. Cellular Heterogeneity in Immune Response

Understanding the full spectrum of cellular responses during infection, including the identification and characterization of specialized immune cell subsets, is crucial to understanding infection outcomes. Xu et al. employed single-cell RNA sequencing to characterize hepatic natural killer (NK) cell heterogeneity during schistosomiasis, identifying distinct cytotoxic NK cell subsets. This study exemplifies how single-cell technologies can reveal previously unrecognized immune cell populations and their specialized functions during parasitic infections.

### 2.3. Economically Important Pathogen Systems

Many pathogens affecting agriculture and aquaculture remain understudied at the molecular level [14,15,16]. However, the molecular basis of disease resistance in crop species and the genetic architecture underlying quantitative trait loci for pathogen resistance need further elucidation. Saxe et al. advanced our understanding of plant–pathogen interactions by identifying co-located quantitative trait loci for vigor and disease resistance in walnut tree (*Juglans microcarpa* × *Juglans regia*) rootstocks. This work provides valuable insights for crop breeding programs and demonstrates the complex genetic architecture underlying multi-trait resistance. Xie et al. conducted comprehensive proteomic and metabolomic analyses of grass carp (*Ctenopharyngodon idella*) infected with grass carp reovirus (GCRV), providing insights into viral pathogenesis in one of the most economically important fish species produced in aquaculture and identifying potential therapeutic targets. Wang et al. performed whole-genome characterization of the cytochrome P450 gene family in *Tilletia horrida*, a fungus that causes significant yield losses in rice around the world, advancing our understanding of fungal pathogenicity mechanisms and metabolic adaptations in plant-pathogenic fungi.

### 2.4. Viral Regulatory Mechanisms

The complex regulatory networks employed by viruses to manipulate host cells, including microRNA-mediated regulation, require in-depth characterization [17]. Prančlová et al. investigated tick-borne encephalitis virus (TBEV) infection in human brain pericytes, revealing robust chemokine production despite weak viral replication, providing insights into neuroinflammation mechanisms during viral encephalitis. Yu et al. characterized pyroptosis induction by rabies virus (RABV) in neuronal cells, advancing our understanding of virus-induced programmed cell death and its role in viral pathogenesis and neurological damage. RABV is a neurotropic virus that causes fatal neurological disease and has an extremely wide host range and wide geographic distribution, making it an important public health problem.

### 2.5. Computational Predictions

Computational screening and network medicine demonstrate how in silico approaches can guide experimental research. Avila-Bonilla and Salas-Benito employed computational screening approaches to predict microRNA targets in flavivirus 3′ UTR genomes, demonstrating how bioinformatics can inform antiviral development strategies by identifying viral regulatory elements. Flavivirus is a genus of the family Flaviviridae, transmitted by arthropod vectors, that includes West Nile virus (WNV), dengue virus (DENV), yellow fever virus (YFV), Japanese encephalitis virus (JEV), TBEV, and several other viruses which lead to extensive morbidity and mortality in humans. Caixeta et al. applied network medicine principles to predict and validate gene expression patterns in human macrophages infected with *Leishmania major*. This computational–experimental approach demonstrates how systems biology can guide experimental design and reveal novel aspects of host–pathogen interactions.

## 3. Future Research Directions

To address current knowledge gaps and develop new tools for combatting infection, several key areas require focused research attention, as exemplified by the studies in this collection and others.

The diverse pathogen systems represented in this collection (viruses, bacteria, parasites, fungi) affecting different hosts (mammals, plants, fish) suggest that cross-kingdom pathogen comparative approaches could reveal universal principles of host–pathogen interactions. The inclusion of plant and aquaculture pathogen systems emphasizes the need for integrated One Health approaches that consider pathogen threats across human, animal, and plant systems. Also, understanding how environmental factors influence host–pathogen interactions will be increasingly important given the expanding range of vector-borne diseases due to climate change [18].

The hormonal modulation of pathogen responses highlights the need for precision medicine. Future research should systematically investigate how sex hormones influence pathogen susceptibility, immune responses, and treatment efficacy across different infectious diseases.

Studies on host–pathogen interactions take advantage of fundamental differences between the biochemistry of hosts and pathogens, identified through research on the interactions at play during infection, and may identify potential therapeutic targets. The underlying mechanisms of protective effects exhibited by naturally occurring host disorders may also help identify new potential targets for therapeutic approaches, helping to delay the development of life-threatening pathogen densities until clearing immunity or the effect of a co-delivered traditional drug are achieved [19,20,21].

Taking novel methodological approaches is crucial to moving forward. Integrating single-cell omics analysis will provide a more comprehensive insights into cellular responses during infection, and the computational approaches represent the beginning of Artificial Intelligence integration in host–pathogen research. Machine learning approaches could accelerate the identification of novel therapeutic targets and predict treatment outcomes.

## 4. Concluding Remarks

As we continue to face emerging infectious diseases, antimicrobial resistance challenges, and the impacts of climate change on pathogen distribution, the molecular insights provided by these studies will be instrumental in developing new diagnostic, therapeutic, and preventive strategies.

This Special Issue reflects a thriving, rapidly evolving field that bridges molecular biology, computational science, and translational medicine. The discoveries highlighted here provide a foundation for future work aiming to translate these fundamental discoveries into practical applications that will benefit human, animal, and plant health.

## Figures and Tables

**Table 1:** List of the articles present in this Special Issue.

First Author	Reference
Nolasco-Pérez, TDJ	Nolasco-Pérez, T.D.J.; Salazar-Castañón, V.H.; Cervantes-Candelas, L.A.; Buendía-González, F.O.; Aguilar-Castro, J.; Le-gorreta-Herrera, M. Testosterone Modulates Oxidative Stress in a Sexually Dimorphic Manner in CBA/Ca Mice Infected with *Plasmodium berghei* ANKA. Int. J. Mol. Sci. 2025, 26, 3898, doi:10.3390/ijms26083898.
Xu, F	Xu, F.; Gao, Y.; Li, T.; Jiang, T.; Wu, X.; Yu, Z.; Zhang, J.; Hu, Y.; Cao, J. Single-Cell Sequencing Reveals the Heterogeneity of Hepatic Natural Killer Cells and Identifies the Cytotoxic Natural Killer Subset in Schistosomiasis Mice. Int. J. Mol. Sci. 2025, 26, 3211, doi:10.3390/ijms26073211.
Saxe, HJ	Saxe, H.J.; Leslie, C.A.; Brown, P.J.; Westphal, A.; Kluepfel, D.A.; Browne, G.T.; Dandekar, A.M. Co-Location of QTL for Vigor and Resistance to Three Diseases in *Juglans microcarpa* × *J. regia* Rootstocks. Int. J. Mol. Sci. 2025, 26, 903, doi:10.3390/ijms26030903.
Xie, J	Xie, J.; Jia, Z.; Li, Y.; Liao, L.; Zhu, Z.; Wang, Y.; Huang, R. Analysis of GCRV Pathogenesis and Therapeutic Measures Through Proteomic and Metabolomic Investigations in GCRV-Infected Tissues of Grass Carp (*Ctenopharyngodon idella*). Int. J. Mol. Sci. 2024, 25, 11852, doi:10.3390/ijms252111852.
Prančlová, V	Prančlová, V.; Hönig, V.; Zemanová, M.; Růžek, D.; Palus, M. Robust CXCL10/IP-10 and CCL5/RANTES Production Induced by Tick-Borne Encephalitis Virus in Human Brain Pericytes Despite Weak Infection. Int. J. Mol. Sci. 2024, 25, 7892, doi:10.3390/ijms25147892.
Yu, D	Yu, D.; Jin, R.; Liu, J.; Zhang, C.; Duan, C.; Luo, X.; Yang, W.; Liu, C.; Liang, J.; Li, X.; et al. Rabies Virus Infection Causes Pyroptosis of Neuronal Cells. Int. J. Mol. Sci. 2024, 25, 5616, doi:10.3390/ijms25115616.
Wang, Y	Wang, Y.; Shi, Y.; Li, H.; Wang, S.; Wang, A. Whole Genome Identification and Biochemical Characteristics of the *Tilletia horrida* Cytochrome P450 Gene Family. Int. J. Mol. Sci. 2024, 25, 10478, doi:10.3390/ijms251910478.
Avila-Bonilla, RG and Salas-Benito, JS	Avila-Bonilla, R.G.; Salas-Benito, J.S. Computational Screening to Predict MicroRNA Targets in the Flavivirus 3′ UTR Genome: An Approach for Antiviral Development. Int. J. Mol. Sci. 2024, 25, 10135, doi:10.3390/ijms251810135.
Caixeta, F	Caixeta, F.; Martins, V.D.; Figueiredo, A.B.; Afonso, L.C.C.; Tieri, P.; Castiglione, F.; De Freitas, L.M.; Maioli, T.U. Expression of Network Medicine-Predicted Genes in Human Macrophages Infected with *Leishmania major*. Int. J. Mol. Sci. 2024, 25, 12084, doi:10.3390/ijms252212084.
